# Editorial: Sensing and control for efficient human-robot collaboration

**DOI:** 10.3389/fnbot.2024.1370415

**Published:** 2024-02-20

**Authors:** Jing Luo, Chao Zeng, Zhenyu Lu, Wen Qi

**Affiliations:** ^1^School of Automation, Wuhan University of Technology, Wuhan, China; ^2^Department of Informatics, Universität Hamburg, Hamburg, Germany; ^3^Bristol Robotics Laboratory, University of the West of England, Bristol, United Kingdom; ^4^School of Future Technology, South China University of Technology, Guangzhou, China

**Keywords:** sensing and control, human-robot collaboration (HRC) system, artificial intelligence-AI, human intelligence (Humint), complex collaborative tasks

Human-robot collaboration (HRC) systems are expected to perform complex collaborative tasks in uncertain environments with advanced sensing and control technologies, which are widely utilized in industry and daily life. These systems are necessary for robots to collaborate with humans efficiently. To achieve this, the control strategies of HRC systems need to be updated based on human intervention by allocating the robot's capabilities and human intelligence for elaborated and complicated tasks. Generally, HRC systems are engaged in interdisciplinary research in terms of intelligent sensing and advanced control and have obtained great achievements. However, the performance of HRC still cannot meet human expectations in real applications. Ultimately, two key problems have to be addressed for HRC system: (1) how to combine sensing and control strategies for more efficient HRC interaction performance; (2) how to integrate learning and artificial intelligence techniques to improve the intelligence of HRC systems for complicated and elaborated tasks. This Research Topic in Frontiers of Neurorobotics aims to bring together the latest studies of HRC systems in terms of theoretical achievements and experimental results from industry and academia. Five manuscripts are accepted after a standard and strict review process in this Research Topic. A brief review of the published articles is given as below.

Wu et al. proposed a reinforcement-learning-based push-grasping method in a cluttered scene with multiple target objects for intelligent manipulation. Compared with previous methods, this algorithm has taken the states of all the targets into consideration to expand the grasping space of all targets for pushing action, improving the grasping efficiency of the whole robotic system in such an unstructured environment. In addition, the authors also utilized the mask fusion method for multiple targets, defined the graspable probability concept and provided the multi-target push-grasping reward mechanism to enhance the robot's abilities of autonomous cognition and decision-making. The proposed method was trained in simulation and then utilized in a real system without retraining or fine-tuning. To improve the synchronization performance of supernumerary robotic limbs (SRL) and minimize the interference influence with the human gait in the process of HRC, Liu et al. developed a method of human-robot synchronized (HRS) walking dynamics based on SRL in relatively ideal working condition. A simulation calculation results were provided by this HRS model for selecting the optimal values of stiffness and damping coefficients between the human and the SRL based on the passive dynamic walking theory. In order to validate the proposed HRS algorithm, a wheel-legged SRL system was designed and the best synchronization results were achieved when only damping units were used in the process of straight-line waling without tuning. In Wang, the authors introduced the Res-FLNet to enhance the robustness and privacy-preserving autonomous driving by using multi-modal sensing and learning control algorithm. In this framework, the features were extracted from visual inputs based on ResNet-50, the sequential dependencies of the multi-modal data were captured through Long short-term memory model. Federated learning was utilized for enabling model training locally without sharing raw data for tackling the privacy problems of individual robots. Meanwhile, the aggregating model was updated from the central sever learning collective knowledge with preserving data privacy among different autonomous vehicles. Mang et al. summarized the newest achievements in brain-computer interface (BCI)-mediated post stroke motor function recovery. In this survey, they rethought the cognitive aspects of BCI-mediated post stroke motor function restoring (PMFR) from the human side. Then, the BCI-mediated PMFR based brain signal discrimination was shown for getting the brain activities. Subsequently, the triggering motor commands and strengthening of external devices were summarized for BCI-mediated PMFR. Next, the multi-modal feedback of closed loop BCI system was reviewed. They finally discussed the future research endeavors: (i) Understanding compensatory motor control mechanisms in post-stroke patients; (ii) Customizing multi-modal feedback for individual patients; (iii) Advancements in BCI-compatible brain stimulation techniques; (iv) Enhancing brain motor signal extraction techniques and devices; and (v) Developing customized soft, wearable exosuits. In Lu et al., a novel system was described for lumbar puncture and epidural steroid injection. A probe was installed in the robotic system, which was used to capture the impedance spectroscopy measures bio-impedance signals. A Bayesian neural network was utilized to classify the soft tissues based on the bio-impedance spectra and determine their categories. The robotic system's motions could been controlled by the master computer based on the recognition results.

Finally, we expect that this topic can provide inspiration for the research on robot learning and control for physical interaction task scenarios.

## Author contributions

JL: Writing—original draft, Writing—review & editing. CZ: Writing—original draft, Writing—review & editing. ZL: Writing—original draft, Writing—review & editing. WQ: Writing—original draft, Writing—review & editing.

